# Supplementary material for: Perceptions of oral health promotion in primary schools among health and education officials, community leaders, policy makers, teachers, and parents in Gulu district, northern Uganda: A qualitative study
Source: PLoS One. 2023 Nov 2;18(11):e0293761. doi: 10.1371/journal.pone.0293761 (PMC10621852; doi:10.1371/journal.pone.0293761)
Supplement: S1 Table — (DOCX) [file pone.0293761.s001.docx]

Table: Overview of themes and subthemes with illustrative quotes

| **Theme** | **Subtheme** | **Illustrative quotes** |
| --- | --- | --- |
| Delivery of oral health promotion | Engagement of health workers, the community, and companies | *“Of course, sometimes there are schools, there are schools that by their own plan they may want such services to be offered to them, they invite us, we go just check their students, give oral talk, may be see a few complaints like that ...”*(Interview 01_Public health dental officer) |
|  |  | *“For oral health, we [school management committee members] talk about keeping the tooth like I told you. You tell them to brush their teeth, or we talk to the teachers so that they tell them the advice, they tell them to brush their teeth at least if not 3 or 2 times a day.”* (Interview 12_Member school management committee) |
|  |  | *“It is good that there is this organisation from “Colgate” has ever supplied our school.”* (Interview 05_Headteacher) |
|  | Skills-based health education | *“I have participated, even in the class I teach and during general assembly.  We started talking about personal hygiene, they should maintain brushing the teeth everybody. We do it in the morning then in the evening as we go to bed. Yes, yes, part of the curriculum you find the issue of personal hygiene is taught right from p1, so in that we can also get some knowledge from the curriculum to help the children.”* (Interview 07_Headteacher) |
|  |  | *“Even we always, when we are for the PE we also teach them. Physical education goes with the oral health.”* (Interview 10_Member PTA) |
|  |  | *“….although to some extent like in classes when teachers are teaching occasionally, they bring in something like that. How we should take care of the tooth from home.”* (Interview 13_Parent) |
|  | Oral health services | *“Normally what we do, we have sessions especially during physical education where the teachers emphasise on the basic health, personal hygiene in which we have the components some minor checking of the mouth…”(Interview 05_Headteacher)* |
|  |  | *“Of course, they have also done this thing of most schools now have got forms for examination.” (Interview 03_Dental surgeon)* |
|  |  | *“Then we also do what we call ART, atraumatic restorative treatment, whereby in early carious lesions when the cavity is not all that deep it can be excavated and you apply what, uhm GI filling material or composite then we also do general conservation….”* (Interview 02_Public health dental officer) |
| Implementation challenges of oral health promotion | Insufficient funding | *“So, oral hygiene there are no partners who are handling that, even the package of health education there are very few partners supporting health education that is why we don’t have that ability to move to radio talk shows frequently or even to go to schools.* “ (Interview 14_City health officer) |
|  |  | *“Much as it [the budget] may not be sufficient, but it is, at least it can do some work.”* (Interview 09_Public health dental officer) |
|  | Unsatisfactory skills-based education | *“Uhm, okay to me I think they are not providing enough skills because actually for you to make someone to do something it should be practical. Yeah, you should show them how to brush their teeth because you can even tell someone that brush your teeth but even that person does not know how to brush…”*(Interview 11_Parent) |
|  |  | *The curriculum is so shallow;  it is just general knowledge.”* (Interview 03_Dental surgeon) |
|  | Inadequate dental screening | *“And then some parents are still so much of having the forms filled in their favour instead of filling the form as per the situation in the mouth.  Not just the parents dodging the cost because when they come, they are against you putting a negative report.”* (Interview 03_Dental Surgeon) |
|  | Poor oral health knowledge | *“What the parents always know like ahh you just maybe you must brush after every meal. That is what, that is the only knowledge they have.”* (Interview 02_Public health dental officer) |
|  |  | *“Simply people don’t have enough information to know that a cavity which is developing you can delay it if you start practicing certain things.”* (Interview 01_Public health dental officer) |
|  |  | *“Uhm, that is why I said that we may not be doing up to the dot. Yes so, the best is the few that we know we do practice from our homes we also transfer to the children, but now going into deep like may be the technical part that one needs maybe somebody who has serious knowledge there also can come in and guide us.”*  (Interview 07_Member PTA) |
|  | Limited parental involvement | *“When you have challenge with any parent you invite the parent others also don’t come [to school]”* (Interview 17_Headteacher) |
|  |  | *“Some of the parents don’t have time for this.”* (Interview 13_Parent) |
| Developing an oral health policy | Lack of oral health policy | *“Generally, what we know is that we always have guides on general health of the school, not specifically on oral health.”*  (Interview 05_Headteacher) |
|  |  | *“We don’t have the policy for oral and dental conditions, it is not there.”*  (Interview 04_District health educator) |
|  | Policies to improve oral health education | *“Yeah, because for me I see this [oral health policy] is a very important component as we go around in teaching and learning process. I would think if there can be a regular kind of monitoring and checks.”* (Interview 05_Headteacher) |
|  |  | *“Maybe you can say that they could create a space, provision for parents, that time for parents to come and talk to the children about those, about the oral health.”*(Interview 11_Parent) |
|  | Improving the paperwork | *“Children should be examined from there then a report given if we really are serious about helping these children, we need to examine these children.”* (Interview 03_Dental Surgeon) |
|  | Parents’ responsibilities | *“…the responsibility of parents should be in that policy that your responsibility is this and if you have not done this is what will be done on you.”* (Interview 08_Headteacher) |
|  |  | *“Yeah, like I said, it [the oral health policy] should emphasise more on the individual learner taking most of the actionable areas which is required to maintain their dental and oral hygiene and parents to ensure when the children wake up in the morning before going to school, they attend to their dental and oral hygiene first before even breakfast.”* (Interview 04_District health educator) |
