# Supplementary material for: Perceptions of oral health promotion in primary schools among health and education officials, community leaders, policy makers, teachers, and parents in Gulu district, northern Uganda: A qualitative study
Source: PLoS One. 2023 Nov 2;18(11):e0293761. doi: 10.1371/journal.pone.0293761 (PMC10621852; doi:10.1371/journal.pone.0293761)
Supplement: Attachment — Submitted filename: PONE-D-23-08537_reviewer_HC.pdf [file pone.0293761.s003.pdf]

PLOS ONE

Perceptions of oral health promotion in primary schools among health and education officials, community leaders, policy makers, teachers, and parents in Gulu district, northern Uganda: a qualitative study

--Manuscript Draft--

**Manuscript Number:**	PONE-D-23-08537
**Article Type:**	Research Article
**Full Title:**	Perceptions of oral health promotion in primary schools among health and education officials, community leaders, policy makers, teachers, and parents in Gulu district, northern Uganda: a qualitative study
**Short Title:**	Oral health promotion in primary schools among key stakeholders in Uganda: a qualitative study
**Corresponding Author:**	Peter Akera<br>Gulu University Faculty of Medicine<br>Gulu, UGANDA
**Keywords:**	Dental caries; primary school; children; community; oral health education.
**Abstract:**	Introduction<br>One in every two cases of caries in deciduous teeth occurs in low- and middle-income countries (LMICs). The aim of the World Health Organisation's (WHO) Healthy Schools Program is to improve the oral health of children. This study explored perceptions of implementation of the Ugandan oral health schools' program in Gulu district, northern Uganda.<br>Methods<br>Semi-structured interviews were conducted with a purposive sample of 19 participants including health and education officials, community leaders, policy makers, teachers, and parents. All interviews were transcribed verbatim and analysed thematically.<br>Results<br>Our study identified three themes: (1) components of oral health promotion, (2) implementation challenges of oral health promotion, and (3) development of an oral health policy. The components of oral health promotion in schools included engagement of health workers, the community, companies, skills-based education, and oral health services. Participants were concerned about insufficient funding, unsatisfactory skills-based education, and inadequate dental screening. Participants reported that there was an urgent need to develop oral health policy to guide implementation of the program at scale.<br>Conclusions<br>Schools provided oral health promotion that aligned with existing features of the WHO's health-promoting school framework. Implementation of this strategy could be enhanced with increased resources, adequate oral health education, and explicit development of oral health policy.
**Order of Authors:**	Peter Akera
	Sean E Kennedy
	Aletta E Schutte
	Robyn Richmond
	Michael Hodgins
	Raghu Lingam
**Additional Information:**	
**Question**	**Response**
**Financial Disclosure**<br><br>Enter a financial disclosure statement that	The author(s) received no specific funding for this work.

Powered by Editorial Manager® and ProduXion Manager® from Aries Systems Corporation

describes the sources of funding for the work included in this submission. Review the submission guidelines for detailed requirements. View published research articles from *PLOS ONE* for specific examples.<br><br>This statement is required for submission and **will appear in the published article** if the submission is accepted. Please make sure it is accurate.<br><br>**Unfunded studies**<br>Enter: *The author(s) received no specific funding for this work.*<br><br>**Funded studies**<br>Enter a statement with the following details:<br>• Initials of the authors who received each award<br>• Grant numbers awarded to each author<br>• The full name of each funder<br>• URL of each funder website<br>• Did the sponsors or funders play any role in the study design, data collection and analysis, decision to publish, or preparation of the manuscript?<br>• **NO** - Include this sentence at the end of your statement: *The funders had no role in study design, data collection and analysis, decision to publish, or preparation of the manuscript.*<br>• **YES** - Specify the role(s) played.<br><br>* typeset	
**Competing Interests**<br><br>Use the instructions below to enter a competing interest statement for this submission. On behalf of all authors, disclose any competing interests that could be perceived to bias this work—acknowledging all financial support and any other relevant financial or non-financial competing interests.<br><br>This statement is required for submission and **will appear in the published article** if the submission is accepted. Please make sure it is accurate and that any funding	The authors have declared that no competing interests exist.

sources listed in your Funding Information later in the submission form are also declared in your Financial Disclosure statement.<br><br>View published research articles from *PLOS ONE* for specific examples.<br><br>**NO authors have competing interests**<br><br>Enter: *The authors have declared that no competing interests exist.*<br><br>**Authors with competing interests**<br><br>Enter competing interest details beginning with this statement:<br><br>*I have read the journal's policy and the authors of this manuscript have the following competing interests: [insert competing interests here]*<br><br>* typeset	
**Ethics Statement**<br><br>Enter an ethics statement for this submission. This statement is required if the study involved:<br><br>• Human participants<br>• Human specimens or tissue<br>• Vertebrate animals or cephalopods<br>• Vertebrate embryos or tissues<br>• Field research<br><br>Write "N/A" if the submission does not require an ethics statement.<br><br>General guidance is provided below. Consult the submission guidelines for detailed instructions. **Make sure that all information entered here is included in the Methods section of the manuscript.**	The study was approved by the research ethics committees of the University of New South Wales (number HC200028) and Gulu University (GUREC-051-20). Written informed consent was obtained from all participants.

**Format for specific study types**<br><br>**Human Subject Research (involving human participants and/or tissue)**<br>• Give the name of the institutional review board or ethics committee that approved the study<br>• Include the approval number and/or a statement indicating approval of this research<br>• Indicate the form of consent obtained (written/oral) or the reason that consent was not obtained (e.g. the data were analyzed anonymously)<br><br>**Animal Research (involving vertebrate animals, embryos or tissues)**<br>• Provide the name of the Institutional Animal Care and Use Committee (IACUC) or other relevant ethics board that reviewed the study protocol, and indicate whether they approved this research or granted a formal waiver of ethical approval<br>• Include an approval number if one was obtained<br>• If the study involved *non-human primates*, add *additional details* about animal welfare and steps taken to ameliorate suffering<br>• If anesthesia, euthanasia, or any kind of animal sacrifice is part of the study, include briefly which substances and/or methods were applied<br><br>**Field Research**<br><br>Include the following details if this study involves the collection of plant, animal, or other materials from a natural setting:<br>• Field permit number<br>• Name of the institution or relevant body that granted permission	
**Data Availability**<br><br>Authors are required to make all data underlying the findings described fully available, without restriction, and from the time of publication. PLOS allows rare exceptions to address legal and ethical concerns. See the PLOS Data Policy and FAQ for detailed information.	Yes - all data are fully available without restriction

A Data Availability Statement describing where the data can be found is required at submission. Your answers to this question constitute the Data Availability Statement and **will be published in the article**, if accepted.<br><br>**Important:** Stating 'data available on request from the author' is not sufficient. If your data are only available upon request, select 'No' for the first question and explain your exceptional situation in the text box.<br><br>Do the authors confirm that all data underlying the findings described in their manuscript are fully available without restriction?	
**Describe where the data may be found in full sentences. If you are copying our sample text, replace any instances of XXX with the appropriate details.**<br><br>• If the data are **held or will be held in a public repository**, include URLs, accession numbers or DOIs. If this information will only be available after acceptance, indicate this by ticking the box below. For example: *All XXX files are available from the XXX database (accession number(s) XXX, XXX.)*.<br>• If the data are all contained **within the manuscript and/or Supporting Information files**, enter the following: *All relevant data are within the manuscript and its Supporting Information files.*<br>• If neither of these applies but you are able to provide **details of access elsewhere**, with or without limitations, please do so. For example:<br><br>*Data cannot be shared publicly because of [XXX]. Data are available from the XXX Institutional Data Access / Ethics Committee (contact via XXX) for researchers who meet the criteria for access to confidential data.*<br><br>*The data underlying the results presented in the study are available from (include the name of the third party*	Data cannot be shared publicly because of restrictions that apply to the availability of these data, which were used under license for the current study. Data are available from the corresponding author upon reasonable request and with permission of UNSW.

and contact information or URL).<br>• This text is appropriate if the data are owned by a third party and authors do not have permission to share the data.<br><br>* typeset	
Additional data availability information:	

Manuscript Click here to access/download;Manuscript;Manuscript.docx

Perceptions of oral health promotion in primary schools among health and education officials, community leaders, policy makers, teachers, and parents in Gulu district, northern Uganda: a qualitative study

Peter Akera[1,3*], Sean E Kennedy[2 ¶], Aletta E Schutte[1,4 ¶], Robyn Richmond[1 ¶], Michael Hodgins[2 ¶], Raghu Lingam[2”]

[1] School of Population Health, University of New South Wales, Sydney, Australia

[2] School of Clinical Medicine, Faulty of Medicine & Health, University of New South Wales, Sydney, Australia

[3] Faculty of Medicine, Gulu University, Gulu, Uganda.

[4] The George Institute for Global Health, Sydney, Australia

*Corresponding author

E mail: peterakera2010@gmail.com; p.akera@student.unsw.edu.au

[¶] These authors contributed equally to this work.

[“] Senior author.

Abstract

Introduction

One in every two cases of caries in deciduous teeth occurs in low- and middle-income countries (LMICs). The aim of the World Health Organisation's (WHO) Healthy Schools Program is to improve the oral health of children. This study explored perceptions of implementation of the Ugandan oral health schools' program in Gulu district, northern Uganda.

Methods

Semi-structured interviews were conducted with a purposive sample of 19 participants including health and education officials, community leaders, policy makers, teachers, and parents. All interviews were transcribed verbatim and analysed thematically.

Results

Our study identified three themes: (1) components of oral health promotion, (2) implementation challenges of oral health promotion, and (3) development of an oral health policy. The components of oral health promotion in schools included engagement of health workers, the community, companies, skills-based education, and oral health services. Participants were concerned about insufficient funding, unsatisfactory skills-based education, and inadequate dental screening. Participants reported that there was an urgent need to develop oral health policy to guide implementation of the program at scale.

Conclusions

Schools provided oral health promotion that aligned with existing features of the WHO’s health-promoting school framework. Implementation of this strategy could be enhanced with increased resources, adequate oral health education, and explicit development of oral health policy.

Keywords

Dental caries, primary school, children, community, oral health education.

Introduction

The burden of oral diseases remains a substantial population health challenge in low- and middle-income countries (LMICs). In 2017, of the 532 million cases of caries in deciduous teeth observed globally, 265 million were in LMICs (1). The high prevalence of caries in deciduous teeth has a negative impact on the quality of life of school children. Children with poor oral health suffer from pain and poor oral health negatively impacts activities such as smiling, sleeping, eating, and school attendance (2, 3).

Public health measures such as promoting good oral hygiene, healthy nutritional and behavioural practices, and education about oral diseases (4) reduce the risk of oral diseases. As outlined in the World Health Organisation's (WHO) Healthy Schools Programme, schools provide an optimum location to deliver health promotion activities, where children can develop personal lifelong skills, healthy attitudes, and healthy behaviours, and thereby reduce the risks of oral disease (5).

The Health Promoting School framework incorporates oral health promotion in schools as an integral part of school activities or the curriculum and this supportive environment can also be a channel for interaction with the community. The framework consists of a range of health promotion strategies to improve oral health. These include providing a safe healthy environment to consider oral health, skills-based education, ~~and~~ access to oral health services, improving health promoting policy and practice, and health of the community and engaging health, education, and community leaders (5-7).

There has been limited research in LMICs on the implementation of oral health promotion in schools. Most research has been on oral health status and the associated risk factors for oral diseases among school children (8-11). In Uganda, one study reported on the impact of establishing four health promoting schools in rural communities ~~in Uganda~~ (12). That study reported a 26% increase in tooth brushing at least once daily and teachers mentioned the benefits of the program such as a greater awareness about health and fewer absences from school due to 'pain'. Key factors identified for success of the program included changes in policy within participating schools, while reported challenges included irregular delivery of supplies. However, that study did not report on factors that increased implementation efficacy nor on factors that would be required to scale up the intervention to a national program.

We explored the views of key school and community stakeholders on oral health promotion in schools in the Gulu district in Uganda. Findings from this study are compared to current policy and practice and the wider implementation literature, and identified gaps that need to be filled to improve oral health promotion activities in schools and enhance rollout of these health promotion interventions. We assessed components of oral health promotion, implementation challenges of oral health promotion and development of an oral health policy to inform policy makers and implementors on how to improve the oral health program, using Uganda as an example.

Methods

~~We conducted~~ semi-structured interviews with health and education officials, community leaders, policy makers, teachers and parents in Gulu District, Uganda. Gulu district is located in the northern part of Uganda about 330 kilometres north of Kampala, the capital city. Over half (53%) of the total population of around 300,000 people are aged between 0 and 17 years. About a quarter, 26% of persons aged 10-17 years are illiterate. Only 21% of households have access to piped water. The burden of disease in Gulu can be related to poverty, limited access to health facilities and schools, illiteracy, and limited access to clean water.

Our study was approved by the research ethics committees of the University of New South Wales (number HC200028) and Gulu University (GUREC-051-20). We obtained written informed consent from all participants.

Interviews with 19 participants were conducted by PA between November 2021 to February 2022. Semi-structured interviews were carried out to gain a deep understanding of the views of health and education officials, community leaders, policy makers, teachers, and parents on the implementation of oral health promotion in schools as they have first-hand knowledge of oral health promotion. The interviews lasted between 15 and 55 minutes. We purposefully chose participants to ensure variation with regard to rural or urban location of school and occupation, to gain a broad understanding of the diverse contributions to oral health promotion. We had access to names of institutions and description of participants by type of occupation that could identify individual participants during or after data collection.

Semi-structured interview guides were developed framed around key aspects of the WHO guidelines on oral health promotion through schools and they were piloted with three participants in order to gain feedback on the appropriateness of the interview prompts (5-7). Prompts were used to explore areas of interest to the participants in greater detail. Questions were amended as new themes emerged. Emerging theme patterns and interpretations, and reflections of the research were recorded in memos and analytical notes. All interviews were conducted in-person in offices where there was privacy. All interviews were digitally recorded after informed consent was gained. We transcribed participants' responses verbatim and accuracy was checked against audio recordings by a person independent of the research team and transcripts were anonymised.

Data collection and analysis happened concurrently to iteratively refine our interview questions based on emerging codes and themes. Data collection continued until data saturation.

Data analysis

Analysis followed transcription of the interviews. Thematic analysis was carried out as described by Terry et al., 2017 (13). This involved familiarisation and coding of data, theme development, reviewing and defining themes, and developing a report of the themes (13). We generated codes by reading the transcripts repeatedly after which we developed candidate themes and refined codes with similar key features. We used a thematic map to identify and understand potential themes. The authors defined and named candidate themes after shaping, clarifying, or even rejecting themes to ensure the themes worked well in relation to the coded

data, the dataset, and the research questions. Analysis included writing an analytic narrative that encased the presented data extracts, providing a theme definition; short summaries of the core idea and meaning of each theme. Data and the report were shared with participants to check meaning and interpretation. Key themes and the analytic framework were shared, discussed, and challenged at meetings with all authors. Finally, key themes were compared to Proctor's implementation outcomes (14) as part of a triangulation of our findings.

Results

Interviews were completed with 19 stakeholders of which six were members of parent-teacher associations (PTA), three members of school management committee (SMC), one city health officer, one dental surgeon, one district health educator, one education officer, three head teachers and three public health dental officers. Members of PTA, SMC, and head teachers were from five different schools located in northern Uganda.

Three inductive themes were developed from the data. The first theme comprised components of oral health promotion and described how oral health promotion strategies were implemented and comprised three subthemes. The second theme was implementation challenges of oral health promotion and comprised five subthemes describing barriers to successful oral health promotion. The third theme related to the development of oral health policy and comprised four subthemes which analysed participants' perceptions of the development of oral health policy. The themes and subthemes are presented in Table 1. Presented quotes are illustrative of the theme.

Table 1: Overview of themes and subthemes with illustrative quotes

**Theme**	**Subtheme**	**Illustrative quotes**
Delivery of oral health promotion	Engagement of health workers, the community, and companies	*"Of course, sometimes there are schools, there are schools that by their own plan they may want such services to be offered to them, they invite us, we go just check their students, give oral talk, may be see a few complaints like that ..."* (Interview 01_Public health dental officer)
		"For oral health, we [school management committee members] talk about keeping the tooth like I told you. You tell them to brush their teeth, or we talk to the teachers so that they tell them the advice, they tell them to brush their teeth at least if not 3 or 2 times a day." (Interview 12_Member school management committee)
		"It is good that there is this organisation from "Colgate" has ever supplied our school." (Interview 05_Headteacher)
	Skills-based health education	*"I have participated, even in the class I teach and during general assembly. We started talking about personal hygiene, they should maintain brushing the teeth everybody. We do it in the morning then in the evening as we go to bed. Yes, yes, part of the curriculum you find the issue of personal hygiene is taught right from p1, so in that we can also get some knowledge from the curriculum to help the children."* (Interview 07_Headteacher)
		"Even we always, when we are for the PE we also teach them. Physical education goes with the oral health." (Interview 10_Member PTA)
		"….although to some extent like in classes when teachers are teaching occasionally, they bring in something like that. How we should take care of the tooth from home." (Interview 13_Parent)
	Oral health services	*"Normally what we do, we have sessions especially during physical education where the teachers emphasise on the basic health, personal hygiene in which we have the components some minor checking of the mouth…"(Interview 05_Headteacher)*
		"Of course, they have also done this thing of most schools now have got forms for examination." (Interview 03_Dental surgeon)
		"Then we also do what we call ART, atraumatic restorative treatment, whereby in early carious lesions when the cavity is not all that deep it can be excavated and you apply what, uhm GI filling material or composite then we also do general conservation…." (Interview 02_Public health dental officer)
Implementation challenges of oral health promotion	Insufficient funding	*"So, oral hygiene there are no partners who are handling that, even the package of health education there are very few partners supporting health education that is why we don't have that ability to move to radio talk shows frequently or even to go to schools. "* (Interview 14_City health officer)
		"Much as it [the budget] may not be sufficient, but it is, at least it can do some work." (Interview 09_Public health dental officer)
	Unsatisfactory skills-based education	*"Uhm, okay to me I think they are not providing enough skills because actually for you to make someone to do*

		something it should be practical. Yeah, you should show them how to brush their teeth because you can even tell someone that brush your teeth but even that person does not know how to brush…" (Interview 11_Parent)
		The curriculum is so shallow; it is just general knowledge." (Interview 03_Dental surgeon)
	Inadequate dental screening	*"And then some parents are still so much of having the forms filled in their favour instead of filling the form as per the situation in the mouth. Not just the parents dodging the cost because when they come, they are against you putting a negative report."* (Interview 03_Dental Surgeon)
	Poor oral health knowledge	*"What the parents always know like ahh you just maybe you must brush after every meal. That is what, that is the only knowledge they have."* (Interview 02_Public health dental officer)
		"Simply people don't have enough information to know that a cavity which is developing you can delay it if you start practicing certain things." (Interview 01_Public health dental officer)
		"Uhm, that is why I said that we may not be doing up to the dot. Yes so, the best is the few that we know we do practice from our homes we also transfer to the children, but now going into deep like may be the technical part that one needs maybe somebody who has serious knowledge there also can come in and guide us." (Interview 07_Member PTA)
	Limited parental involvement	*"When you have challenge with any parent you invite the parent others also don't come [to school]"* (Interview 17_Headteacher)
		"Some of the parents don't have time for this." (Interview 13_Parent)
Developing an oral health policy	Lack of oral health policy	*"Generally, what we know is that we always have guides on general health of the school, not specifically on oral health."* (Interview 05_Headteacher)
		"We don't have the policy for oral and dental conditions, it is not there." (Interview 04_District health educator)
	Policies to improve oral health education	*"Yeah, because for me I see this [oral health policy] is a very important component as we go around in teaching and learning process. I would think if there can be a regular kind of monitoring and checks."* (Interview 05_Headteacher)
		"Maybe you can say that they could create a space, provision for parents, that time for parents to come and talk to the children about those, about the oral health." (Interview 11_Parent)
	Improving the paperwork	*"Children should be examined from there then a report given if we really are serious about helping these children, we need to examine these children."* (Interview 03_Dental Surgeon)
	Parents' responsibilities	*"…the responsibility of parents should be in that policy that your responsibility is this and if you have not done this is what will be done on you."* (Interview 08_Headteacher)
		"Yeah, like I said, it [the oral health policy] should emphasise more on the individual learner taking most of the actionable areas which is required to maintain their dental and oral hygiene and parents to ensure when the children wake up in the morning before going to school, they attend to their dental and oral hygiene first before even breakfast." (Interview 04_District health educator)

Theme 1: Components of oral health promotion

The theme, 'Components of oral health promotion' describes the different oral health promotion activities reported by stakeholders. The components of oral health promotion were categorised according to the WHO Health Promoting School's framework which comprised providing a safe healthy environment to consider oral health, skills-based education, and access to oral health services, improving health promoting policy and practice, and health of the community and engaging health, education, and community leaders (5-7).

Engagement of health workers, the community, and companies

Routine outreach to schools was conducted by dental health professionals where they provided oral health education, screened children's teeth for decay and treated children for oral diseases and conditions. The outreach was based on quarterly plans prepared by health workers. "Outreach clinics" were attended by children, teachers, and community members.

> *"….basically, from the health facility we arrange (school oral health screening), inform the schools then they mobilise the parents, inform them of what is going to happen at school. We screen and then treat those we can treat."* (Interview 09_Public health dental officer)

Members of the SMC and PTA were engaged in this process. Occasionally the members of SMC and PTA in conjunction with teachers provided oral health education to children in class on brushing of teeth. Parents were also engaged in the program which involved sensitisation of oral hygiene of their children.

> *"….at school you find that the SMC and the PTA come to school, they get the problem at school level as they go out there, they will be what, ambassadors*

to tell the community, the parents, that you know, you need to allow your children [to] brush their teeth, rinse teeth and also do flossing." (Interview 08_Teacher)

Some schools had yearly programs with toothpaste companies to promote oral health. The activities of the program included distribution of toothpaste and toothbrushes, how-to-brush demonstrations, and oral health awareness. Teachers and students actively participated in the program. This was seen as part of the company's corporate social responsibility.

"….there is also a time we had a program with "Colgate". They gave us several toothpaste and toothbrushes so whenever we could go to those schools…." (Interview 02_Public health dental officer)

Skills-based health education

The provision of theoretical and practical oral health education was integrated in regular education by teachers and was provided according to the primary school curriculum. Topics included human health, toothbrushing skills, the importance of brushing, and consequences of not brushing such as tooth decay. Teaching methods include lectures, demonstrations, and practical experiences. The curriculum was delivered by teachers who had received pre- and in-service training on oral health promotion from while at teacher training colleges and non-governmental organisations (NGOs), respectively.

"We are following the curriculum in health education especially in the issue of the health of the teeth. It is one of the topics that we teach especially in p4 (children aged 10 years). The teaching is done in a classroom and practically

the learning aids are given or prepared by the teacher for example we have some samples of toothpaste and toothbrushes.” (Interview 08_Teacher)

Oral health services

Children were screened by teachers during health parades and physical exercise to identify dental caries, poor oral hygiene, signs of oral disease including halitosis, tooth colour change, and other oral health complaints as part of other general health education and care programs. Children were referred by teachers to nearby health facilities for the management of oral health conditions. Some schools required children to have routine medical assessments by different specialists such as opticians, dentists, and ear, nose, and throat surgeons and present a medical report.

“In some more modern schools when the children are going back for holidays, they give them some medical forms whereby each of them is supposed to go and see a different specialist.” (Interview 02_Public health dental officer)

Health workers from nearby facilities carried out tooth extractions and atraumatic restorative treatment (ART) during school outreach programs.

Theme 2: Implementation challenges of oral health promotion

Implementation challenges were assessed as limitations to delivery of oral health promotion in schools. Participants described challenges including insufficient

funding, unsatisfactory skills-based education, inadequate dental screening, poor oral health knowledge, and limited parental involvement.

Insufficient funding

Participants perceived that oral health was not prioritised by schools and health facilities. This was evidenced by what participants viewed as an insufficient budget for activities and a limited number of NGOs implementing oral health interventions. Limited resources affected the way the schools and health facilities were able to deliver oral health education, the number of school outreached by health workers, and the ability of professionals to treat oral diseases.

> *"And since people don't take it [oral health] as a priority, it is always underbudgeted for. …....because of limited resources we end up reaching few schools…."* (Interview 14_City health officer)

Unsatisfactory skills-based education

Many teachers and health workers felt that oral health education at school was inadequate because the school curriculum only provides general information. There were limited practical sessions, and a lack of knowledge and skills around oral health. In addition, there is limited in-service training of teachers on oral health education and promotion. There was a lack of educational materials such as charts, posters, and toothbrushes for demonstration of brushing.

> *"In the curriculum we have health education, normally these teachers do not concentrate so much on those particular areas they just give general information."* (Interview 05_Headteacher)

Inadequate dental screening

School staff lacked knowledge in identification, prevention, and treatment of oral diseases. A few schools required children to have periodic assessments from specialist doctors, but dental screening was not among the conditions assessed.

> *"In the biodata form there is a provision that shows the allergy thing, maybe the child is suffering from this, should be stipulated by a doctor. About the oral health? Ah no. We only do for the chronic diseases and then we have included the COVID also."* (Interview 07_Headteacher)

For schools that required children to be screened by a dentist the information collected was not used by schools for management of cases. It was suggested that medical assessment of children each term was not working because parents did not want to admit to ill health of their children in case it was somehow detrimental to the child's school progress or reflected badly on them as parents.

> *"That one [filling medical forms] is not working because as a parent I have got a good school and I want my child to go to that school, so people always go to a health practitioner they know. So, it is just filled as demanded. But in real sense the child could be having so many dental caries, so people try to hide so that they get access to the school. So that one can only work if it is done from schools but not from home."* (Interview 14_City health officer)

Poor oral health knowledge

Participants mentioned that the community lacked information around practices to delay development of tooth decay. Information was limited to tooth brushing after meals, but it was reported that parents and children did not know how long a person is supposed to brush their teeth. The population perceived tooth

extractions as the only treatment for oral disease and only sought oral health care when they had problems.

> *"Most cases if not 90 then 100 percent of dental patients they go to the hospital when they already have problem, none of them will really take their time to go for a check-up."* (Interview 02_Public health dental officer)

Participants also felt that teachers lacked knowledge on providing oral health education.

> *"For us who are imparting the knowledge are also unskilled in that field."* Interview 17_Headteacher)

Limited parental involvement

Teachers mentioned that parents did not educate their children on oral hygiene or enforce regular cleaning of their children's teeth in the morning before they left for school. Health workers reported that when they conducted school outreach sessions , parents generally did not attend. Teachers found that when parents were invited to the school to discuss health issues concerning their children, they would often not attend, which in many cases was due to lack of resources or competing demands such as they had to work. Participants mentioned parents spend little time with their children, have limited time for oral health promotion and don't care about their children's oral health as this was not viewed as a priority compared to the day-to-day hardships.

> *"Actually we are supposed to meet parents during parents meeting, so generally we talk to parents about a number of issues including the health of their children but unfortunately the type of parents, either because of the economic level that everybody focuses in looking for money, you find they don't even care."* (Interview 05_Headteacher)

Theme 3: Developing an oral health policy

We assessed the development of policy to guide implementation of oral health promotion in schools. Assessment encompassed policy decisions and actions on factors that affect oral health promotion such as sensitisation of school staff and regular monitoring of the teaching. This guide stakeholders to implement activities to improve oral health of children.

Lack of oral health policy

Participants mentioned that even though they were trying to implement the Healthy Schools Programme, there was no school-based government oral health policy to guide teachers and health workers. A few noted that the policy that children are required to come to school with toothpaste and toothbrushes was not sufficient if it was not supported by oral health education.

"*We don't have the policy for oral and dental conditions, it is not there."* (Interview-04_District Health Educator)

Policies to improve oral health education

Participants felt the need to develop oral health policy to guide them with implementation of activities to improve on oral health of children. They felt that the oral health policy should encompass sensitisation of school staff, regular monitoring of the teaching and learning processes and involvement of parents and oral health professionals.

"….career guidance day could be organised like once a month to have a health talk, they can introduce, they can maybe get a dentist or some other

person in the field to go and sensitise them more." (Interview 02_Public health dental officer)

Improving the paperwork

Respondents felt a practical way of improving oral health was to use the oral health school-based clinics for termly screening of children for oral disease. Health workers said the filling of medical forms in private clinics and public health facilities for termly screening was not working because parents would look for health workers they know and have the medical forms filled stating there was no illness present. Health workers said that this method of screening for oral disease did not provide accurate information about the oral health status of the child. A participant mentioned that during the school term children present with advanced stages of disease and yet the medical forms had recently indicated no problems only a short time before.

"So that is another [policy] area that they should work on that they really need to emphasise that the forms should actually be filled from school not from the clinics when the nurse is there so that we get the true picture, and the child is helped." Interview 03_Dental Surgeon)

Parents' responsibilities

Participants felt it was essential to increase the responsibility of parents for the oral health of their children though this could be problematic due to the needs of the family and difficulties in the household in areas such as finance. However, an explicit "contract" between the school and the family could increase oral health provision in homes.

"Yeah in the oral health policy the key aspect that could be included in the policy which is implementable would be the aspect of brushing, we could

emphasize that each parent takes the responsibility of ensuring that the child's oral health is okay by providing the basic necessities that is required." (Interview 09_Public health dental officer)

Discussion

This study documented the perceptions of key stakeholders, including those with responsibility for implementing an oral health promotion program in a low-income African setting. Previous research has described various implementation interventions for oral health promotion that resulted in positive oral health outcomes (15-17). Yet there has been limited work conducted to understand how best to implement oral health promotion programs in schools. Our study explored the components of oral health promotion such as skills-based health education, implementation challenges like insufficient funding, unsatisfactory skills-based education, and inadequate dental screening and development of policy to improve oral health education, paperwork, and parents' responsibilities.

Previous quantitative and mixed methods research identified facilitators for oral health promotion in schools which were capacity and availability of human resources, support and advocacy, and the presence of a policy framework. While the barriers were time constraints, limited involvement, large classes, lack of adequate resources, funding, and collaboration at the local level (18, 19). While the participants reported many positives in the delivery of this program such as children are taught to use locally available materials to brush their teeth, funding for school outreach, and the presence of persons in charge of health in schools, there were several barriers to implementation that potentially could have hampered program

uptake and success. From our thematic analysis we considered our findings in relation to four of Proctor's key implementation outcomes (14): acceptability, fidelity, appropriateness, and feasibility.

In general, the components of an oral health program were accepted by stakeholders. Strategies to improve oral health of school children involve interventions that require the engagement of all the stakeholders that we interviewed, including teachers, health and education managers, parents, and community leaders (4, 5). Participants described good engagement from schoolteachers, parents, and one external organisation. Also, participants commended the preventative nature of the programme as a benefit for the community.

In terms of fidelity, we found that participants delivered oral health promotion in alignment with the WHO health promoting school framework with some limitations.[5, 6] Interventions designed according to the WHO's framework have previously resulted in positive oral health outcomes (15-17, 20). However, insufficient funding and a lack of clear policy guidance for the school proved a challenge to the fidelity of the program. Other studies have similarly noted these implementation challenges (12,18). A WHO global survey of school-based health promotion reported inadequate finances, inadequate policy framework, and lack of collaboration between local level as implementation challenges (18). Other challenges included lack of high-level leadership and governance, poor awareness, attitude, and support among local users, and failure to provide quality services (18). In other areas of Uganda, a qualitative study among teachers and key stakeholders of four health

promoting schools reported irregular delivery of supplies and a low number of children who participated in the program (12).

Regarding appropriateness, our study demonstrates that delivery of oral health promotion in schools created awareness of oral health for children and the community, children were screened, referred, and treated; children received toothpaste and toothbrushes; and there was active participation by children and teachers. This replicated previous work that reported the benefits from becoming a health-promoting school were greater knowledge and awareness about health, active participation in activities to promote oral health, fewer absences from school due to ‘pain’ or the need for emergency dental treatment, pride among children and created awareness among community members (12).

The extent to which the oral health program was feasible varied according to participants’ reports. While some participants felt that the lack of resources and poor oral health knowledge would hinder oral health promotion, the program engaged the community and companies, provided skills-based education, and oral health services. Our findings indicated the presence of planned outreach initiatives to schools, and to organisations like SMC and PTA for community engagement, collaboration with private companies, a focussed school curriculum and willingness of children to participate in screening, could contribute to successful implementation of such programs. Partnerships, political commitment, timely and accurate communication of information, and evidence-based interventions are necessary for public health programs to succeed (21).

Further research is needed to assess other key implementation issues specifically adoption, sustainability, and penetration of oral health promotion in schools (14). In addition, as we identified a lack of funding which was repeatedly raised as a barrier to success, an analysis of marginal cost, cost-effectiveness, and cost-benefit would be important for program implementation (14).

The national oral health policy proposes a school oral health program that emphasises implementation of oral health education, screening, and training of teachers in oral health education (22). However, the national oral health care policy of 2007 is outdated, as it was written over ten years ago. The need for data to inform development of oral health policy has been expressed by researchers and health planners (8, 23).

Future interventions to promote oral health in schools should consider these challenges to implementation. The implication of this study is that health and education officials, community leaders, policy makers, teachers and parents' perceptions of oral health promotion were aligned with existing local national policy and features of the WHO's health-promoting school framework. However, there is need to provide adequate resources and develop policy for promoting oral health among school children.

The strengths and limitations of this study

The first author (PA) is a dental surgeon and public health specialist. He has over 20 years' experience in management of oral conditions and oral health promotion in northern Uganda. His knowledge and subject knowledge could have

impacted on the questions asked during the interview and how he viewed the data collected. However, as per best practice, he kept a log of his thoughts and feelings during and after the interviews. In addition, themes were discussed with the wider multidisciplinary study team that were non-dental in training.

Health and education officials, community leaders, policy makers, teachers, and parents were experts in their respective fields with several years of experience. This might have influenced how they answered questions towards the perceived "ideal answers" rather than what they had experienced. However, we purposefully selected a diverse population and report on prominent and unusual themes therefore the study delivers a deep understanding on the views of participants sampled. In addition, purposeful sampling of participants in Gulu District limits generalisability of our findings to other settings in Uganda. Further limitations are that head teachers may have had bias in providing the list of candidate members PTA and SMC.

Conclusions

Health and education officials, community leaders, policy makers, teachers and parents play a critical role in program planning, evidence-based decision making and allocation of resources, and ultimately the success of public health interventions. Schools provided oral health promotion that aligned with existing features of the WHO's health-promoting school framework, however a lack of resources, training and practical policy limit the scope and effectiveness of interventions. To improve oral health in the region and other low resource settings, there is need to address implementation challenges such as inadequate resources for oral health promotions and develop oral health policy.

Acknowledgements

The authors are grateful to the key stakeholders who participated in the study. We appreciate cooperation of Gulu district and Gulu City administration during the study. We thank the late Professor Mark J Obwolo contributing to the design of the study and for his guidance on data collection.

References

1. Bernabe E, Marcenes W, Hernandez CR, Bailey J, Abreu LG, Alipour V, et al. Global, Regional, and National Levels and Trends in Burden of Oral Conditions from 1990 to 2017: A Systematic Analysis for the Global Burden of Disease 2017 Study. Journal of Dental Research. 2020;99(4):362-73.
2. Mashoto KO, Åstrøm AN, David J, Masalu JR. Dental pain, oral impacts and perceived need for dental treatment in Tanzanian school students: a cross-sectional study. Health and Quality of Life Outcomes; BMC Central. 2009;7(73).
3. Jurgensen N, Petersen PE. Oral health and the impact of socio behavioural factors in a cross-sectional survey of 12-year-old school children in Laos. . BMC Oral Health 2009;9(29).
4. World Health Organization. Promoting oral health in Africa: prevention and control of oral diseases and noma as part of essential noncommunicable disease intervention.2016. ISBN: 978-929023297-1
5. World Health Organization. Oral Health Promotion: An Essential Element of a Health-Promoting School Geneva, Switzerland WHO; 2003. Available from: https://www.who.int/oral_health/media/en/orh_school_doc11.pdf.
6. World Health Organization. Health Promoting Schools: An effective approach to early action on Non-Communicable Disease risk factors Geneva, Switzerland. World Health Organization. 2017. Available from: https://apps.who.int/iris/bitstream/handle/10665/255625/WHO-NMH-PND-17.3-eng.pdf?sequence=1.
7. World Health Organization. Local Action Creating Health Promoting Schools. 2000. Available from:

https://apps.who.int/iris/bitstream/handle/10665/66576/WHO_NMH_HPS_00.3.pdf?sequence=1.

8. Kutesa A, Kasangaki A, Nkamba M, Muwazi L, Okullo I, Rwenyonyi CM. Prevalence and factors associated with dental caries among children and adults in selected districts in Uganda. African Health Sciences. 2015;15(4):1302-7.

9. Muwazi LM, Rwenyonyi CM, Tirwomwe FJ, Ssali C, Kasangaki A, Nkamba ME, et al. Prevalence of oral diseases/conditions in Uganda. African Health Sciences. 2005 5 (3):227-33.

10. Rwenyonyi CMM, L. M. & Buwembo, W. Assessment of factors associated with dental caries in rural communities in Rakai District, Uganda. Clinical Oral Investigations. 2011;15 75–80.

11. Batwala V, Mulogo EM, Arubaku W. Oral health status of school children in Mbarara, Uganda. African Health Sciences. 2007;7(4):232-8.

12. Macnab A, Kasangaki A. 'Many voices, one song': a model for an oral health programme as a first step in establishing a health promoting school. Health Promotion International. 2012;27(1):63-73.

13. Terry G, Hayfield N, Clarke V, Braun V. The SAGE Handbook of Qualitative Research in Psychology. 2017 2021/08/18. London: SAGE Publications Ltd. Available from: https://methods.sagepub.com/book/the-sage-handbook-of-qualitative-research-in-psychology-second-edition.

14. Proctor E, Silmere H, Raghavan R, Hovmand P, Aarons G, Bunger A, et al. Outcomes for implementation research: conceptual distinctions, measurement challenges, and research agenda. Administration and Policy in Mental Health. 2011;38:65-76.

15. Petersen PE, Peng B, Tai B, Bian Z, Fan M. Effect of a school-based oral health education programme in Wuhan City, Peoples Republic of China. International Dental Journal. 2004;54(1):33-41.

16. Peng B, Petersen PE, Bian Z, Tai B, Jiang H. Can school-based oral health education and a sugar-free chewing gum program improve oral health? Results from a two-year study in PR China. Acta Odontologica Scandinavica. 2004;62(6):328-32.

17. Tai BJ, Jiang H, Du MQ, Peng B. Assessing the effectiveness of a school-based oral health promotion programme in Yichang City, China. Community Dentistry and Oral Epidemiology. 2009;37(5):391-8.

18. Jürgensen N, Petersen PE. Promoting oral health of children through schools- Results from a WHO global survey 2012. Community Dental Health 2013;30:204-18.

19. Reddy M, Singh S. The promotion of oral health in health-promoting schools in KwaZulu-Natal Province, South Africa. South African Journal of Child Health. 2017;11(1):16-20.

20. Akera P, Kennedy SE, Lingam R, Obwolo MJ, Schutte AE, Richmond R. Effectiveness of primary school-based interventions in improving oral health of children in low- and middle-income countries: a systematic review and meta-analysis. BMC Oral Health. 2022;22(1):264.

21. Frieden TR. Six components necessary for effective public health program implementation. American Journal of Public Health. 2014;104(1):17-22.

22. Republic of Uganda. Ministry of Health. National Oral Health Policy In: Health, editor. Kampala 2007.

23. Republic of Uganda. Ministry of Health. Health Sector Strategic Plan II 2005/6 - 2009/10. In: Ministry of Health, editor. Kampala, Uganda Ministry of Health 2005.
