# Supplementary material for: Perceptions of oral health promotion in primary schools among health and education officials, community leaders, policy makers, teachers, and parents in Gulu district, northern Uganda: A qualitative study
Source: PLoS One. 2023 Nov 2;18(11):e0293761. doi: 10.1371/journal.pone.0293761 (PMC10621852; doi:10.1371/journal.pone.0293761)
Supplement: Attachment — Submitted filename: Response to reviewers.docx [file pone.0293761.s004.docx]

# Point-by-point response to reviewer comments for the journal PLOS One Manuscript ID PONE-D-23-08537 Akera *et al.*

**Reviewer comments**

Table 1 identifies this theme as “delivery of oral health promotion”

Thank you for your comment. We have changed the text in Table one to include “components of oral health promotion”.

This should de dental pain.

We agree with your comment and have included the word dental in the text (Line number 89). The text now reads ……‘dental pain’. Key factors identified for success of the program included changes in…..

Replace “We” with “This study….” (Line 95)

We have replaced “We” with “This study”. (Line number 95)

Are these objectives of the study (Line number 96-98)

The statement “Findings from this study are compared to current policy and practice and the wider implementation literature, and identified gaps that need to be filled to improve oral health promotion activities in schools and enhance rollout of these health promotion interventions” does not define objectives of the study. We have deleted the statement from the manuscript.

OR Are these the objectives of your study? These read here as objectives of the study, whereas in the results section of the study you state that these were the key themes that were generated from the interviews with the participating stakeholders.

could you please clarify the objectives of the study. (Line number 99-103)

Thank you for your observation. The text presented are the key themes from the study. We have deleted this text from the last paragraph on page. For clarification on the objectives of the study, we have changed the last paragraph on page 5 (Section Introduction) to: This study explored the views of key school and community stakeholders on oral health promotion in schools in the Gulu district in Uganda.

Rewrite this text to read something like: 'Semi-structured interviews were conducted with health and education officials, community leaders, policy makers, teachers and parents in the Gulu District, Uganda'. (Line number 108, 109)

We agree with your suggestion and have rewritten the text in this section to: 'Semi-structured interviews were conducted with health and education officials, community leaders, policy makers, teachers and parents in the Gulu District, Uganda'.

Replace 'Our' with 'This'. (Line 117)

We agree with your comment and have replaced “Our” with “This”.

Re-write this sentence as follows: 'Written consent was obtained for all participants'. (Line number 118,119)

We agree with your suggestion and have re-written the text (Line number 119) to: Written informed consent was obtained for from all participants.

Do not use the personal pronoun in your writing. Re-write the text to avoid the use of 'we'.(Line number 126)

Thank you for your comment. We have changed the text in paragraph 3 on page 6 (Section Methods) to: Participants were purposefully chosen to ensure variation with regard to rural or urban………

Do not use the personal pronoun in your writing. Re-write the text to avoid the use of 'we'. (Line number 128)

We agree that we need to re-write the text to avoid the use of “we” and have changed text in paragraph 3 on page 6 (Section Methods) to: The district health and education officers provided names of schools and health facilities during physical……

How did you get access to the names of institutions? What kind of institutions did you approach? and how did you approach them, ie, via email, phone call, etc? Who within the institution did you approach to approve recruitment of participants from their institution? (Line number 129)

We agree that this would be helpful to the reader and have added the following text to Paragraph 3 on Page 6-7 (Section Methods): The district health and education officers provided names of schools and health facilities during physical meetings and phone calls. Head teachers and health facility in-charges provided approval for recruitment of participants from their institutions. The names of institutions and description of participants by type of occupation could identify individual participants during or after data collection.

How did you determine that you achieved data saturation?

We have included text in the last paragraph on Page 7 (Section Methods) explaining how data saturation was determined. Data saturation was reached where no new themes emerged from data analysis; this was discussed with all authors.

Did you determine data saturation for each of the different stakeholders (ie, health and education officials, community leaders, policy makers, teachers, and parents) or did you determine data saturation across all stakeholders.

Data saturation was determined across all stakeholders. We have included this information in the last paragraph on Page 7 (Section Methods). Data saturation was reached where no new themes emerged from data analysis; this was discussed with all authors.

Are you aware of the following editorial regarding data saturation in qualitative studies:

Thorne S. The great saturation debate: what the 'S Word' means and doesn't mean in qualitative research reporting. Canadian Journal of Nursing Research 2020, Vol 52 (1) 3-5. reporting.

We are aware of the editorial regarding data saturation and have changed the last paragraph on Page 7 (Section Methods) to: Data saturation was reached where no new themes emerged from data analysis; this was discussed with all authors.

How did you determine that you reached data saturation from each of these stake holders when you only interviewed one member from each of these stakeholder groups?

We agree however we looked for common themes across stakeholder groups. We agree that if we were doing qualitative sub analysis by group(which we were not) we would need greater numbers in each subgroup. We have added this to our discussion section. We have changed the last paragraph on page 21 (Section Discussion) to: Common themes were looked for across all stakeholders and not sub analysis by group, this would need greater numbers in each subgroup. Further limitations are that head teachers may have had bias in providing the list of candidate members PTA and SMC.

How were each of these from five different schools located in northern Uganda, when you only interviewed three SMCs and three head teachers?

Six participants (3 SMC and 3 headteachers) were purposefully selected from five different schools.

Were these three themes developed from your data or were they based on the objectives of your study?

The themes were iteratively developed from the data. In the last paragraph on page 5 were clarify that “This study explored the views of key school and community stakeholders on oral health promotion in schools in the Gulu district in Uganda.”

In table 1, theme one is referred to as 'Delivery of Oral Health Promotion'

We have changed to “components of oral health promotion” in table 1.

I suggest that these quotes be included in the body of the text rather than in Table 1 as is presented here. In the body of the text you include other quotes that are not included in this table. It would be far more meaningful to the reader to have all the quotes included in the relevant sections of the text.

We agree it would be more meaningful to the reader if illustrative quotes are included in relevant sections of the text. In table 1, we had included additional quotes for the relevant sections on top of what is included in the text. We have deleted table 1 and maintain the quotes in the relevant sections. We have not included text from table one in the text as each section would have more than one quote.

In table 1 this is presented as ' Delivery of Oral Health promotion' (Line 184)

We have excluded table 1 from the manuscript.

See previous comment. (Line 185)

We have excluded table 1 from the manuscript.

The word 'describes' should be replaced with 'comprises. (Line 185)

We have replaced the word “describes” with “comprises”. Paragraph

This is confusing. Were the subthemes of the 'components of health promotion' theme categorised according to the 'WHO Health promoting school framework' or according to the health promotion activities reported by the stakeholders who participated in this study. Please clarify.

The themes were identified according to the health promotion activities reported by stakeholder and categorised according to the 'WHO Health promoting school framework'.

Need to make it clear in the text that this is a subtheme.

We have included the word “subtheme” to identify all subthemes in the text. For example, we have changed Paragraph 2 on Page 12 (Section Results) to: Subtheme 1: Engagement of health workers, the community, and companies and Paragraph 5 on Page 13 (Section Results) to: Subtheme 2: Skills-based health education.

'was reported to be conducted...' (Line number 193)

We have changed the text to 'was reported to be conducted...'(Paragraph 4 on Page 12)

'was reported to be based...' (line number 195)

We have changed the text to 'was reported to be based...'

'were reported to be attended...' (Line number 196)

We have changed the text to 'were reported to be attended...'

'Some schools were reported to have yearly programs..' (Line number 211)

We have changed paragraph 4 on page 13 (Section Subtheme 1: Engagement of health workers, the community, and companies) to: Some schools were reported to have yearly programs with toothpaste companies to promote oral health.

I would not mention the actual name of the company in a publication. Maybe replace the word 'Colgate'with an 'external oral health care organisation' (Line number 216)

Thankyou. We have replaced the word “Colgate” with 'external oral health care organisation' (Paragraph 4 on page 13) “….*there is also a time we had a program with “an external oral health care organisation”. They gave us several toothpaste and toothbrushes so whenever we could go to those schools….”* (Interview 02_Public health dental officer)

Make it clear that this is a subtheme (Line number 219)

We have changed the heading of the subtheme to: Subtheme 2: Skills-based health education (The last paragraph on page 13).

'was reported to be integrated....' (Line number 220)

In the last paragraph on page 13 we have changed the text to: ….was reported to be integrated.

was reported to be provided...' (Line number 221)

In the last paragraph on page 13 we have changed the text to: ……. and was reported to be provided...'

'was noted to be delivered..' (Line number 225)

In the last paragraph on page 13 we have changed the text to: ……. The curriculum was noted to be delivered by teachers who…………

Make it clear that this is a subtheme (Line number 234)

We have changed the text in the first paragraph on page 14 (Section Results)to: Subtheme 3: Oral health services

Replace with: 'It was reported by the participants that children were screened ...(Line number 235)

We have replaced the text in the first paragraph on page 14 (section Results) with: 'It was reported by the participants that children were screened ...

'Health workers from nearby facilities were reported to carry out tooth extractions...' (Line number 247)

We have changed the text in the last paragraph on page 14 (Section Results) to: Health workers from nearby facilities were reported to carry out tooth extractions……

'School staff were considered by the participants to lack knowledge in identification...' (Line number 279)

We have changed the text in the first paragraph on page 16 (Section Results) to: School staff were considered by participants to lack knowledge in identification,……

'...the information collected was reported that it was not used by schools for management of cases.' (Line number 286-287)

We have changed the second paragraph on page 16 (Section Results) to: For schools that required children to be screened by a dentist, it was reported that the information collected was not used by schools for management of cases

Subtheme? (Line number 297)

We have changed the first paragraph on page 17 (Section Results) to: Subtheme 4: Poor oral health knowledge.

Subtheme? (Line number 311)

We have changed the last paragraph on page 17 (Section Results) to: Subtheme 5: Limited parental involvement

Subtheme? (Line number 341)

We have changed the last paragraph on page 18 (Section Results) to Subtheme 2: Policies to improve oral health education.

'...develop an oral health policy..' (Line number 342)

We have changed the first paragraph on page 19 (Section Results) to: Participants felt the need to develop an oral health policy to guide them with implementation of activities to improve on oral health of children.

replace 'on' with 'the' (Line number 343)

We have replaced 'on' with 'the' . The first paragraph on page 19 now reads: Participants felt the need to develop an oral health policy to guide them with implementation of activities to improve the oral health of children.

what exactly do you mean by 'sensitisation' in this context? what would that involve for the school staff? (Line number 344)

By sensitisation we mean providing oral health education. We have changed the first paragraph on page 19 (Section Results) to: They felt that the oral health policy should encompass oral health education of school staff, regular monitoring of the teaching and learning processes and involvement of parents and oral health professionals.

What does the participant mean by 'sensitise' in this context? (Line number 349)

By sensitisation we mean providing oral health education. We have changed the quote in the first paragraph on page 19 (Section Results) to: “….*career guidance day could be organised like once a month to have a health talk, they can introduce, they can maybe get a dentist or some other person in the field to go and sensitise (provide health education) them more.”* (Interview 02_Public health dental officer)

Subtheme? (Line number 351)

We have changed the last paragraph on page 19 (Section Results) to: Subtheme 3: Improving the paperwork

Subtheme? (Line number 365)

We have changed the text to: Subtheme 4: Parents’ responsibilities

Again this sounds like an aim or an objective of the study, and not a finding from the study. (Line number 382-383)

We agree with your comment and have changed the first paragraph on page 20 (Section Discussion) to: Our study identified the components of oral health promotion such as skills-based health education, implementation challenges like insufficient funding, unsatisfactory skills-based education, and inadequate dental screening and development of policy to improve oral health education, paperwork, and parents’ responsibilities.

Which participants? the participants of this study or studies referred to at the start of this paragraph (ie. references 18 and 19)? (Line number 393)

Here we refer to participants of our study and have changed paragraph 2 on page 21 (Section Discussion) to: ). ……..While the participants of this study reported many positives ………..

Which program are your referring to here? the program discussed in reference 18 and 19? (Line number 393)

Here we refer to oral health program in Gulu district and have changed paragraph 2 on page 21 (Section Discussion) to: ). While the participants of this study reported many positives in the delivery of this program in Gulu district such as children are taught to use……………..

Are you referring here to the components of oral health promotion program identified in your research? (Line number 401)

Yes, we are referring to the components of the oral health promotion program identified in our research. We have changed paragraph 3 on page 21 (Section Discussion) to: In general, the components of an oral health program this research identified were accepted by stakeholders. Strategies to improve oral health of school children involve interventions that require the engagement of all the stakeholders…

Replace 'programme' with 'program'. you need to be consistent in your spelling of the word 'program' throughout you manuscript. (Line number 407)

We have replaced 'programme' with 'program'. Paragraph 3 on page 21 (Section Discussion) now reads: ……..Also, participants commended the preventative nature of the program as a benefit for the community.

'frameworks' (Line number 417)

We have changed the text to frameworks. Paragraph 4 on page 22 reads: A WHO global survey of school-based health promotion reported inadequate finances, inadequate policy frameworks, …….

between 'local level' what? (Line number 418)

Here we mean local level stakeholders and have changed paragraph 4 on page 22 to: …….A WHO global survey of school-based health promotion reported inadequate finances, inadequate policy frameworks, and lack of collaboration between local stakeholders as implementation challenges (18).

'..with the existing local...' (Line number 463)

We have changed the last paragraph on page 23 (Section Discussion) to: The implication of this study is that health and education officials, community leaders, policy makers, teachers and parents’ perceptions of oral health promotion were aligned with the existing local national policy and features of the WHO’s health-promoting school framework.

this link does not work. (Line number 526-527)

We provide a link to the document that works and have changed the reference list to:

5. World Health Organization. (2003). Oral Health Promotion: An Essential Element of a Health-Promoting. World Health Organization. Available from: https://apps.who.int/iris/handle/10665/70207

this link also does not work. Line number

We provide a link to the document that works and have changed the reference list to:

6. World Health Organization. (2017). Health Promoting Schools: An effective approach to early action on Non-Communicable Disease risk factors. World Health Organization. Available from: https://www.who.int/publications/i/item/health-promoting-school-an-effective-approach-for-early-action-on-ncd-risk-factors

this link does not work. (Line number 535-536)

We provide a link to the document that works and have changed the reference list to:

7. World Health Organization. (2000). Local Action Creating Health Promoting Schools. World Health Organization. Available from: https://www.who.int/publications/i/item/local-action-creating-health-promoting-schools

this link is not correct. (Line number 554-555)

We have removed the link from the reference list and provide a DOI and an Online ISBN instead.

13. Terry G, Hayfield N, Clarke V, Braun V. The SAGE Handbook of Qualitative Research in Psychology. 2017 2021/08/18. London: SAGE Publications Ltd. ISBN: 9781526405555. DOI: https://dx.doi.org/10.4135/9781526405555
