# Supplementary material for: Perceptions of oral health promotion in primary schools among health and education officials, community leaders, policy makers, teachers, and parents in Gulu district, northern Uganda: A qualitative study
Source: PLoS One. 2023 Nov 2;18(11):e0293761. doi: 10.1371/journal.pone.0293761 (PMC10621852; doi:10.1371/journal.pone.0293761)
Supplement: Attachment — Submitted filename: Response to reviewers.docx [file pone.0293761.s005.docx]

# Point-by-point response to reviewer comments for the journal PLOS One Manuscript ID PONE-D-23-08537R1 Akera *et al.*

**Reviewer comments**

I can understand the reviewers' confusion with regards to the way you have presented some of your themes and subthemes given you made it very clear you conducted inductive analysis, and yet at places you alluded to deductive categorisation. I would like the authors to further address the below:

1. Clearly explain in methods under data analysis how you analyse your data - which part is inductive and when do you apply deductive processes, for instance, using the WHO framework.

Thank you for your comment. We have clearly explained in the methods section under data analysis that the analysis was inductive and changed the text in line 145 to: An inductive approach was used for data analysis after transcription of the interviews.

2. Please re-address the reviewer comment in the 1st paragraph under Theme 1 - "This is confusing. Were the subthemes of the 'components of health promotion' theme categorised according to the 'WHO Health promoting school framework' or according to the health promotion activities reported by the stakeholders who participated in this study. Please clarify." It is not enough just to clarify in your response, you should edit the text directly to make it clearer.

We agree with your comment and have deleted the sentence stating the 'components of health promotion' theme categorised according to the 'WHO Health promoting school framework'. We have changed the first paragraph under theme 1 to: The theme, ‘components of oral health promotion’ comprises the different oral health promotion activities reported by stakeholders and comprised engagement of health workers, the community and companies, skills-based education, and oral health services.

3. Please address the reviewer comment in the 1st paragraph under Theme 3 - "This is confusing. did you actively seek out information on the development of policies or was this a topic that was raised by the participants. In other words, was 'the assessment of the development of policies to guide the implementation of oral health promotion in schools' an objective of this study or was it a finding from the interview transcriptions?" You have missed this.

We agree that the 1^st^ paragraph under theme 3 is confusing. The need for policy development was raised by participants and was a finding from the interview and have changed the paragraph to: Participants mentioned the need for the development of policy to guide implementation of oral health promotion in schools. They mentioned policy decisions and actions on factors that affect oral health promotion such as sensitisation of school staff and regular monitoring of the teaching.

4. I appreciate the authors' deletion of Table 1 in response to the reviewers' comments. However, I would prefer table 1 to be attached either as an appendix or as "supplementary materials" and referred to within text. This would help to demonstrate the depth and breath of the data collected

Thank you for the comment. We have provided table 1 as supplementary material and referred to the table within the text. We have changed line 175-176 to: The themes and subthemes are presented in supplementary file s1. Presented quotes are illustrative of the theme.
